# Association of Plasma Magnesium with Prediabetes and Type 2 Diabetes Mellitus in Adults

**DOI:** 10.1038/s41598-017-13050-7

**Published:** 2017-10-06

**Authors:** Sijing Chen, Xiaoling Jin, Jun Liu, Taoping Sun, Manling Xie, Wei Bao, Xuefeng Yu, Xuefeng Yang, Yan Zhang, Haibo Zhang, Zhilei Shan, Liegang Liu

**Affiliations:** 1 Department of Nutrition and Food Hygiene, Hubei Key Laboratory of Food Nutrition and Safety, School of Public Health, Tongji Medical College, Huazhong University of Science and Technology, Wuhan, 430030 China; 2 Ministry of Education Key Lab of Environment and Health, School of Public Health, Tongji Medical College, Huazhong University of Science and Technology, Wuhan, 430030 China; 3 Department of Epidemiology, College of Public Health, University of Iowa, Iowa City, IA 52242 USA; 4 Division of Endocrinology, Department of Internal Medicine, Tongji Hospital, Tongji Medical College, Huazhong University of Science and Technology, Wuhan, 430030 China; 5 Hubei Provincial Key Laboratory of Yeast Function, Yichang, 443003 China

## Abstract

Our study aimed to assess the associations of plasma magnesium with prediabetes and type 2 diabetes (T2D) among Chinese adults. We conducted a case-control analysis of 4447 participants: 867 newly diagnosed prediabetes patients, 1475 newly diagnosed T2D patients and 2105 normal glucose tolerance (NGT) individuals. In a subsample of 599 apparently healthy individuals, we measured plasma hs-CRP levels to examine their relation to plasma magnesium. Plasma magnesium and hs-CRP were measured by inductively coupled plasma mass spectrometry and an enzyme-linked immunosorbent assay, respectively. Plasma magnesium decreased from NGT to prediabetes to T2D, and was inversely associated with prediabetes and T2D. After adjustment for potential confounding factors, the ORs from the lowest to highest quartiles of plasma magnesium were 1, 0.57 (95%CI, 0.44–0.74), 0.49 (0.37–0.65) and 0.51 (0.37–0.70) for prediabetes, and 1, 0.26 (0.20–0.33), 0.15 (0.12–0.20) and 0.15 (0.11–0.20) for T2D. Consistently, plasma magnesium was inversely correlated with plasma hs-CRP in our subsample analysis; the geometric mean hs-CRP concentration for ascending quartiles of plasma magnesium were 1.29 (1.06–1.57), 1.16 (0.95–1.41), 1.00 (0.81–1.22), and 0.71 (0.58–0.88) mg/l. Plasma magnesium was independently and inversely associated with prediabetes and T2D in Chinese adults.

## Introduction

Magnesium is an essential mineral found in many foods, including whole grains, green leafy vegetables, coffee, legumes and nuts^1^. Meanwhile, magnesium is an essential cofactor for multiple enzymes involved in glucose metabolism and is hypothesized to play a role in glucose homeostasis, insulin action and in the development of type 2 diabetes (T2D)^2^. Prediabetes, characterized by impaired fasting glucose (IFG) and/or impaired glucose tolerance (IGT), is considered an important risk factor for the development of overt diabetes and cardiovascular disease^3,4^. Epidemiological studies have investigated the association of serum magnesium with prediabetes and T2D, but the findings were inconsistent^5–9^. On the other hand, recent studies indicated that increasing dietary magnesium intake may be associated with a reduced risk of T2D and have a dose-response relationship^10,11^. In addition, calcium has been suggested having a role in the development of T2D for its potential in improving pancreatic β-cell function and insulin sensitivity^12–16^, and dietary calcium and magnesium intakes are moderately/highly correlated in diverse populations^5,15–17^. However, few studies were conducted to regard the associations of serum or plasma magnesium with prediabetes and T2D, considering the confounding of plasma calcium within the same population.

Therefore, the objective of this study was to investigate the relationship between plasma magnesium concentrations and newly diagnosed prediabetes and T2D in a large case-control study conducted among a Chinese population. To explore possible mechanisms, we also examined whether plasma magnesium was associated with plasma high sensitivity C-reactive protein (hs-CRP) concentrations in a subsample of apparently healthy individuals.

## Results

### Patient characteristics

Anthropometric and metabolic characteristics of the 4447 participants (867 prediabetes, 1475 T2D, 2105 NGT) are shown in Table 1. Plasma magnesium concentrations were significantly decreased in the individuals with prediabetes and T2D compared with the controls (median: 22.12 mg/l in NGT, 21.42 mg/l in prediabetes, and 19.84 mg/l in T2D, *P* < 0.001). Compared to controls, the patients with prediabetes and T2D had higher BMI, prevalence of family history of diabetes and hypertension, higher levels of total cholesterol, triglyceride, fasting plasma glucose (FPG), fasting plasma insulin (FPI), 2-h post-glucose load, HbA1C, lower plasma calcium and magnesium concentration. When looking at the insulin sensitivity indexes, we observed higher HOMA-IR in prediabetes and T2D.

**Table 1:** Anthropometric and metabolic characteristics of NGT, prediabetes and T2D groups.

Variable	NGT	Prediabetes	T2D	*P* value
	n = 2105	n = 867	n = 1475	
Age (years)	52.21 ± 12.89	52.96 ± 12.22	51.20 ± 11.23	<0.001
Male (%)	59.71	61.15	59.78	0.754
BMI (kg/m^2^)	23.30 ± 3.53	25.09 ± 5.19	25.14 ± 3.93	<0.001
Smoker (%)	35.11	27.16	33.60	<0.001
Drinker (%)	34.39	32.54	35.08	0.480
Family history of diabetes (%)	8.32	17.30	22.62	<0.001
Hypertension (%)	22.89	33.50	35.78	<0.001
FPG (mmol/l)	5.09 ± 0.66	6.16 ± 0.53	9.72 ± 3.21	<0.001
OGTT2h (mmol/l)	6.43 ± 0.95	8.74 ± 1.46	17.61 ± 5.56	<0.001
HbA1C (%)	5.57 ± 0.40	5.94 ± 0.60	8.79 ± 2.37	<0.001
FPI (mmol/l)	7.88 (5.38–11.45)	9.54 (6.58–13.98)	9.46 (6.14–14.35)	<0.001
HOMA-β	85.87 (59.54–122.59)	73.94 (49.63–113.47)	36.15 (19.07–62.75)	0.067
HOMA-IR	1.90 (1.26–2.76)	2.58 (1.76–3.84)	3.84 (2.53–6.01)	<0.001
Total cholesterol (mmol/l)	4.39 (3.57–5.14)	4.69 (3.87–5.42)	4.61 (3.98–5.32)	<0.001
Triglyceride (mmol/l)	1.12 (0.8–1.57)	1.30 (0.89–1.86)	1.38 (0.95–2.11)	<0.001
HDL-c (mmol/l)	1.25 (0.98–1.54)	1.21 (0.98–1.5)	1.11 (0.89–1.4)	<0.001
LDL-c (mmol/l)	2.58 (2.04–3.19)	2.74 (2.1–3.34)	3.02 (2.36–3.67)	0.550
Mg (mg/l)	22.12 ± 2.78	21.42 ± 2.97	19.84 ± 3.47	<0.001
Ca (mg/l)	73.14 ± 9.56	71.10 ± 9.83	68.60 ± 11.28	<0.001

Abbreviations: BMI, body mass index; FPG, fasting plasma glucose; FPI, fasting plasma insulin; HDL-c, high-density lipoprotein cholesterol; HOMA-β, homeostasis model assessment of β cell function; HOMA-IR, homeostasis model assessment of insulin resistance; LDL-c, low-density lipoprotein cholesterol; NGT, normal glucose tolerance; OGTT2h, 2-h post-glucose load; T2D, type 2 diabetes. Data were presented as percentage for categorical data, mean (standard deviation) for parametrically distributed data or median (interquartile range) for nonparametrically distributed data.

### Association of serum magnesium levels with prediabetes and T2D

**Figure 1:**
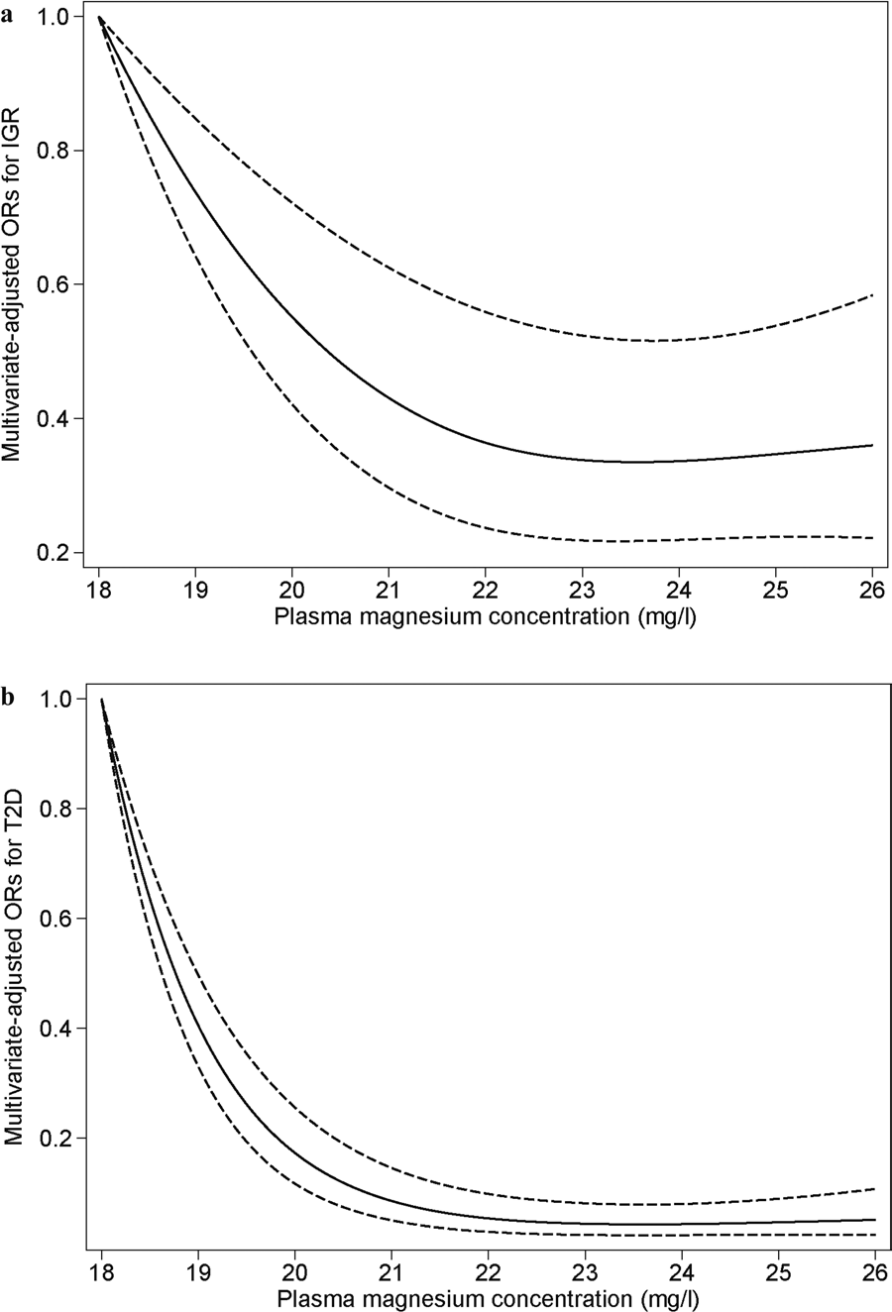
Adjusted ORs for prediabetes and T2D by plasma magnesium concentrations. Lines represent ORs (solid line) and 95% CIs (dashed line) based on restricted cubic splines for plasma magnesium concentrations. The analysis was adjusted for age, sex, BMI, current smoking status, current alcohol drinking status, family history of diabetes, hypertension and plasma calcium. (**a**) Adjusted ORs for prediabetes; (**b**) Adjusted ORs for T2D.

Table 2 presents odds ratios (ORs) for prediabetes and T2D associated with the levels of plasma magnesium concentrations as continuous variables and categorized into quartiles according to the distribution in the controls. Lower ORs for prediabetes and T2D were associated with higher plasma magnesium concentrations. After adjustment for age, sex, BMI, current smoking status, current alcohol drinking status, hypertension, family history of diabetes, and plasma calcium, the ORs (95% confidence interval (CI)) for prediabetes and T2D in the highest quartile compared with the lowest quartile of plasma magnesium were 0.51 (0.37–0.70) and 0.15 (0.11–0.20), *P* for trend < 0.001. Meanwhile, the adjusted ORs for prediabetes and T2D across 1 mg/l higher plasma magnesium were 0.90 (95% CI, 0.86–0.94) and 0.76 (0.73–0.79). In spline regression models, the odds of prediabetes and T2D decreased significantly with increasing magnesium concentrations at less than 22 mg/l, and no significant decline thereafter (Fig. 1). The nonlinear spline terms were not statistically significant (*P* > 0.05), indicating that the relationship between plasma magnesium concentration and T2D was nonlinear.

**Table 2:** Associations of plasma magnesium concentration with prediabetes and T2D.

Variables	Quartiles of plasma magnesium concentration (mg/l)				Per 1 mg/l of plasma magnesium	*P* value for trend
	1 (Lowest)	2	3	4 (Highest)		
	≤20.47	20.48–22.01	22.02–23.65	≥23.65		
**Prediabetes** ***vs*** *.* **NGT**						
Model 1	1	0.56 (0.44–0.70)	0.44 (0.34–0.56)	0.44 (0.35–0.57)	0.88 (0.86–0.91)	<0.001
Model 2	1	0.55 (0.43–0.71)	0.46 (0.36–0.60)	0.47 (0.37–0.61)	0.89 (0.86–0.93)	<0.001
Model 3	1	0.57 (0.44–0.74)	0.49 (0.37–0.65)	0.51 (0.37–0.70)	0.90 (0.86–0.94)	<0.001
**T2D** ***vs*** *.* **NGT**						
Model 1	1	0.30 (0.25–0.36)	0.17 (0.14–0.22)	0.19 (0.16–0.24)	0.79 (0.77–0.82)	<0.001
Model 2	1	0.29 (0.23–0.36)	0.18 (0.14–0.22)	0.19 (0.15–0.24)	0.80 (0.77–0.82)	<0.001
Model 3	1	0.26 (0.20–0.33)	0.15 (0.12–0.20)	0.15 (0.11–0.20)	0.76 (0.73–0.79)	<0.001

Model 1, adjusted for age, sex and BMI. Model 2, adjusted for Model 1, current smoking status, current alcohol drinking status, family history of diabetes, and hypertension. Model 3, adjusted for Model 2 and plasma calcium.

By stratifying data according to age, sex, BMI, family history of diabetes and hypertension, the inverse associations were consistently observed in all subgroups except the <50 years group for prediabetes (Table 3). Interaction effects were found between plasma magnesium concentration and sex (*P* < 0.001), age (*P* < 0.001), as well as between plasma magnesium concentration and hypertension (*P* = 0.031) for T2D; however, the reason for such interactions remains to be elucidated.

**Table 3:** Adjusted ORs for each 1 mg/l increment of plasma magnesium associated with prediabetes and T2D in subgroups.

Groups	%	Prediabetes	*P* for interaction	%	T2D	*P* for interaction
**Sex**						
Male	59.7	0.91 (0.86–0.97)	0.710	60.1	0.75 (0.71–0.79)	<0.001
Female	40.3	0.86 (0.80–0.93)		39.9	0.75 (0.70–0.80)	
**Age**						
<50	40.3	0.99 (0.91–1.06)	0.060	37.4	0.83 (0.78–0.88)	<0.001
≥50	59.7	0.87 (0.82–0.92)		62.6	0.72 (0.68–0.76)	
**BMI**						
<24	51.7	0.89 (0.83–0.95)	0.650	54.5	0.73 (0.68–0.77)	0.075
≥24	48.3	0.91 (0.85–0.96)		45.5	0.77 (0.73–0.82)	
**Family history of diabetes**						
Yes	14.4	0.87 (0.77–0.98)	0.233	11.1	0.73 (0.65–0.83)	0.534
No	85.6	0.88 (0.84–0.93)		88.9	0.76 (0.73–0.79)	
**Hypertension**						
Yes	27.9	0.91 (0.84–0.99)	0.792	25.9	0.80 (0.74–0.86)	0.031
No	72.1	0.89 (0.84–0.94)		74.1	0.73 (0.70–0.77)	

Models adjusted for age, sex, BMI, current smoking status, current alcohol drinking status, family history of diabetes, hypertension, and plasma calcium. The % in 2nd column represents the percent of each subgroup in the populations of prediabetes and NGT. The % in 5th column represents the percent of each subgroup in the populations of T2D and NGT.

### Association of serum magnesium levels with hs-CRP

There was a consistent relation between hs-CRP levels and plasma magnesium in a subsample of 599 apparently healthy individuals study (Table 4). After adjustment for sex, age, BMI, hypertension and family history of diabetes, the geometric mean hs-CRP concentration for ascending quartiles of plasma magnesium were 1.31 (1.08–1.59), 1.17 (0.96–1.42), 0.99 (0.81–1.22), and 0.70 (0.57–0.86) mg/l. Although plasma calcium is highly correlated with plasma magnesium (correlation coefficient = 0.68, *P* < 0.001), further adjustment for plasma calcium did not attenuate the association between plasma magnesium and hs-CRP concentrations and the geometric mean hs-CRP concentration for ascending quartiles of plasma magnesium were 1.29 (1.06–1.57), 1.16 (0.95–1.41), 1.00 (0.81–1.22), and 0.71 (0.58–0.88) mg/l. All the tests for liner trend in every model were significant (*P* < 0.001).

**Table 4:** Associations of inflammatory marker (CRP) with plasma magnesium levels in 599 apparently healthy individuals.

Plasma CRP (mg/l)	Quartiles of plasma magnesium concentration (mg/l)				*P* value for trend
	1 (Lowest)	2	3	4 (Highest)	
Median of plasma magnesium	18.85	20.86	22.43	25.07	
Crude^*^	1.21 (0.81–2.38)	0.99 (0.59–1.74)	0.95 (0.50–1.55)	0.66 (0.32–1.33)	<0.001
Model 1	1.38 (1.19–1.59)	1.11 (0.96–1.29)	0.94 (0.81–1.09)	0.67 (0.58–0.78)	<0.001
Model 2	1.31 (1.08–1.59)	1.17 (0.96–1.42)	0.99 (0.81–1.22)	0.70 (0.57–0.86)	<0.001
Model 3	1.29 (1.06–1.57)	1.16 (0.95–1.41)	1.00 (0.81–1.22)	0.71 (0.58–0.88)	<0.001

CRP, C-reactive protein. ^*^Data were presented as median (interquartilerange). Data were presented as adjusted geometric means (95% CI, unless noted otherwise). Model 1, adjusted for age, sex and BMI. Model 2, adjusted for Model 1, current smoking status, current alcohol drinking status, family history of diabetes, and hypertension. Model 3, adjusted for Model 2 and plasma calcium.

## Discussion

In this study, after adjustment for potential confounding factors, the odds of prediabetes and T2D decreased significantly with increasing magnesium concentrations at less than 22 mg/l, and then no significant decline was observed. The inverse association remained consistent across different subgroups. Also, our subsample study in healthy subjects showed a consistent inverse association between plasma magnesium and plasma levels of hs-CRP.

In our study, the inverse association of plasma magnesium with prediabetes and T2D were both significant after multivariable adjustment. Our findings are generally consistent with results from some of the previous studies^5–8^, which showed lower serum magnesium in diabetic subjects than controls. However, none of the study contained more than 140 diabetic participants or reported a detailed dose-response analysis. Besides, the Atherosclerosis Risk in Communities (ARIC) study found an inverse association between serum magnesium levels and risk of T2D among white participants, but not among black participants^9^. Moreover, even among the white participants, the liner trend between serum magnesium levels and T2D could disappear after all multivariable adjustments. The inconsistent findings for the correlation of serum magnesium with diabetes may attribute to the difference in population, measurements of magnesium and diagnosis of diabetes. These studies were mainly conducted in Western populations that have been shown to have higher insulin resistance than Asians, and diagnosis of diabetes in these studies was different. In this study, as concerns of ORs for T2D, we found a significant interaction between plasma magnesium and sex and age. The interactions have not been assessed in previous studies and may warrant confirmation in further studies.

Our finding of an inverse association between plasma magnesium and hs-CRP levels was consistent with some previous studies. So far, findings on the association of magnesium and CRP levels are not consistent. Several cross-sectional studies reported an inverse association between dietary magnesium intake and CRP levels^18–21^. However, a cross-sectional study found no association between dietary magnesium intake and CRP levels^22^. Nevertheless, a recent meta-analysis based on cross-sectional studies and intervention studies indicated that dietary magnesium intake was inversely associated with serum CRP levels^23^. CRP, as a more sensitive and robust marker of systemic inflammation than other inflammatory markers^24^, has been proved as an independent predictor of risk for the development of T2D^25,26^. The inverse association between magnesium and C-reactive protein suggested that magnesium deficiency might be involved in the development of low chronic inflammatory syndrome, which can modulate diabetes^27^. These findings support the possible pathway for altering systemic inflammation and may explain, at least in part, the potential beneficial effect of high magnesium status on diabetes risk.

Several mechanisms, including facilitating insulin secretion and alleviating inflammation, have been proposed to explain the effect of plasma magnesium on diabetes pathogenesis^28,29^. Over 300 enzymes require the presence of magnesium ions for their catalytic action, including all enzymes utilizing or synthesizing adenosine triphosphate (ATP), and several enzymes that play an important role in glucose metabolism^1,30^. For example, intracellular magnesium deficiency may lead to decreasing tyrosine kinase activity of insulin receptors and to a post-receptorial impairment in insulin action, which may result in the development of insulin resistance^2^. Consistent with the effect of magnesium on insulin resistance, studies have found an inverse association of magnesium intake with fasting insulin level^31^, and HOMA-IR^20,32^, and Hruby *et al*. observed that magnesium intake was particularly beneficial in offsetting risk of developing diabetes among those at high risk^33^. Moreover, experimental findings showed that magnesium modulates cellular events involved in inflammation, because the molecular basis for inflammatory response is probably the result of modulation of intracellular calcium concentration, and magnesium could act as a natural calcium antagonist^34,35^. Consistently, an inverse association of CRP levels with magnesium intake or serum magnesium was reported in several epidemiological studies^18–21^, which suggested that magnesium deficiency might be involved in the development of low chronic inflammatory syndrome, which can modulate T2D^25^. Clinical trials have also suggested that magnesium supplementation downgraded inflammations in healthy or diabetic participants^36–38^, and could be effective in reducing plasma fasting glucose levels in people with diabetes and improving insulin-sensitivity parameters in people at high risk of diabetes^39^.

Our subjects with T2D were confined to the newly diagnosed and drug naive in order to exclude the effects of artificial interventions, because diabetes patients customarily take many measures to control blood glucose levels, and drugs can change the status of metabolism including magnesium^1^. Moreover, we defined diabetes based mainly on fasting and postprandial glucose levels from an oral glucose tolerance test (OGTT), and in our study, we tried to adjust for confounding factors influencing the association between plasma magnesium and diabetes, including plasma calcium.

Our study has several limitations. First, the cross-sectional nature of our study does not allow us to infer any causality between plasma magnesium and T2D, and plasma magnesium level may be reduced by the development of T2D^40^. Therefore, these findings should be confirmed in further prospective cohort studies. Second, our measurement of magnesium was confined to plasma compartment, accounting for roughly 1% of whole body magnesium level. Nonetheless, it has been suggested that if one with suspected magnesium deficiency, a low serum magnesium concentration is sufficient to confirm the diagnosis^29^. We used plasma magnesium concentration as a biomarker to measure magnesium status to avoid possible bias through dietary assessment, such as systematic measurement error in diet exposure and the influence of other nutrients on the bioavailability of magnesium^41,42^. In addition, all participants in this study were of Chinese Han ethnicity, which minimizes the confounding effects by ethnic background but doesn’t allow us to explore whether the association between plasma magnesium and diabetes is different between ethnicities.

In conclusion, this study demonstrates that plasma magnesium is independently and inversely associated with plasma hs-CRP and odds of newly diagnosed prediabetes and T2D. Further studies are warranted to confirm our findings and elucidate the mechanisms behind this association.

## Methods

### Study population

The study population consisted of 4447 participants: 867 newly diagnosed prediabetes, 1475 newly diagnosed T2D patients and 2105 normal glucose tolerance (NGT) individuals. The patients of newly diagnosed prediabetes and T2D were consecutively recruited from those for the first time attending the outpatient clinics of Department of Endocrinology, Tongji Medical College Hospital, Wuhan, China. Age- and sex-matched healthy NGT individuals were recruited from an unselected population undergoing a routine health check-up in the same hospital from 2007 to 2009. The inclusion criteria of NGT, newly diagnosed prediabetes and T2D were: age ≥30 years, body mass index (BMI) < 40 kg/m^2^, no history of a diagnosis of diabetes, not receiving pharmacological treatment for hyperlipidaemia or hypertension, and not taking medication known to affect glucose tolerance or insulin secretion. Patients with clinically significant neurological, endocrinological or other systemic diseases, as well as acute illness and chronic inflammatory or infective diseases, were excluded from the study. All the participants enrolled were of Chinese Han ethnicity.

This study was conducted in accordance with the Declaration of Helsinki and was approved by the ethics committee of the Tongji Medical College. Written informed consent was obtained from each participant.

### Assessment of prediabetes and T2D

The definitions of prediabetes and T2D met the respective diagnostic criteria recommended by the World Health Organization in 1999^43^. Prediabetes was defined as impaired fasting glucose (FPG ≥ 6.1 mmol/l and <7.0 mmol/l and 2-h post-glucose load <7.8 mmol/l) and/or impaired glucose tolerance (FPG < 7.0 mmol/l and 2-h post-glucose load ≥7.8 mmol/l and <11.1 mmol/l). T2D was diagnosed when FPG ≥ 7.0 mmol/l and/or 2-h post-glucose load ≥11.1 mmol/l.

### Body composition and blood parameters

Personal information on demography was collected by using questionnaires, including sex, age, history of diseases (hypertension and hyperlipemia), family history of diabetes, current smoking status, and alcohol drinking status. Anthropometric data including height (m) and weight (kg) were measured with standardized techniques. BMI was calculated as weight divided by the square of height (kg/m^2^). Blood samples were collected in all participants after an overnight fast of at least 10 hours. All participants were given a standard 75 g glucose solution, and plasma glucose was measured at 0 and 2 hours after administration during the OGTT. FPI, total cholesterol, triglyceride, high-density lipoprotein cholesterol, and low-density lipoprotein cholesterol was measured within 2 hours, as described in our previous study^44^. Homoeostasis model assessment insulin resistance (HOMA-IR) score was computed using the following formula: FPI [m-units (milliunits)/l] × FPG (mmol/L)/22.5. The index of HOMA of β-cell function (HOMA-β) was calculated as (20 × FPI)/(FPG − 3.5)^45^.

### Measurement of plasma magnesium concentrations and hs-CRP

Plasma magnesium concentrations were measured in the Ministry of Education Key Laboratory of Environment and Health at School of Public Health at Tongji Medical College of Huazhong University of Science and Technology using inductively coupled plasma mass spectrometry (ICP-MS) (Agilent 7700 Series, Japan). Plasma samples were stored at −80 °C, and thawed on ice. Working standard solutions were prepared by dilution of 1,000 mg/l calibration verification standard (Agilent P/N 5183–4682). For quality assurance, the CRM (certified reference material) ClinChek No. 8883 and No. 8884 human plasma controls were used. For No. 8883, we determined a concentration of: 17.0 ± 1.1 mg/l (certified: 16.9 ± 1.7 mg/l) and for No. 8884 we measured: 31.1 ± 1.7 mg/l (certified: 29.7 ± 3.0 mg/l). Quality control was performed (1 out of 20 samples), and the inter-assay and intra-assay coefficients of variation were <5% and <5%, respectively. Hs-CRP concentration was measured via high-sensitivity enzyme-linked immunosorbent assay.

### Statistical analysis

Descriptive statistics were calculated for all demographic and clinical characteristics of the study subjects. To test for differences of characteristics among different glucose regulation status, continuous variables were compared using one-way ANOVA, and a Chi-square test was used for categorical variables. For calculation of the ORs for prediabetes and T2D, plasma magnesium concentrations were as continuous variables and categorized in quartiles according to the NGT group: category 1, ≤20.47 mg/l; category 2, 20.48–22.01 mg/l, category 3, 22.02–23.65 mg/l and category 4, ≥23.65 mg/l. Multinomial logistic regression analysis was used to assess the associations of prediabetes and T2D with plasma magnesium concentrations. ORs were adjusted for known risk factors for prediabetes and T2D, including age, sex, BMI, current smoking status (yes or no), and alcohol drinking (yes or no), hypertension, family history of diabetes and plasma calcium concentrations. Tests of linear trend across increasing quartiles of plasma magnesium were conducted by assigning the means of plasma magnesium in quartiles as continuous variables. Nonlinear relationships were examined using restricted cubic splines, excluding values outside the 5th and 95th percentiles via Stata version 12 (Stata Corp).

Then stratified analyses were conducted determining the ORs of prediabetes and diabetes with each 1 mg/l increment of plasma magnesium by age, sex, BMI, current smoking status, and alcohol drinking status, hypertension, and family history of diabetes. Likelihood ratio test was used to determine the interactions between the above variables and plasma magnesium. We examined the association between plasma magnesium concentration and plasma hs-CRP levels. A logarithmic transformation was used to improve the normality of hs-CRP distributions. Multiple linear regression models were used to control for the same potential confounding factors included in the logistic regression model. All data analyses were performed with SAS 9.1 (SAS Institute Inc. Cary, NC, USA). P values presented are two-tailed with a significant level at 0.05.
